# Targeting neurodegeneration and inflammation with thioredoxin-mimetic peptides

**DOI:** 10.1016/j.redox.2025.103899

**Published:** 2025-10-17

**Authors:** Daphne Atlas

**Affiliations:** Dept. of Biological Chemistry, Institute of Life Sciences, The Hebrew University of Jerusalem, Jerusalem, 91904, Israel

**Keywords:** Oxidative stress, MAPK, Neuroinflammation, COPD, Covid-19, Parkinson's disease, Myocardial infarction, mTBI, Platelets aggregation, Radiation, Aging

## Abstract

Neurodegenerative and degenerative disorders are in part, driven by imbalances in cellular inflammatory and oxidation-reduction (redox) states. This vulnerability triggers the activation of highly selective and tightly regulated cellular defense networks against oxidative stress (OS), primarily involving the glutathione (GSH) and the thioredoxin (Trx) enzymatic complexes. These systems operate through reversible oxidation/reduction reactions involving the thiol groups of cysteine (Cys) residues, maintaining redox homeostasis and protecting cells from oxidative damage. To reinforce this defense under pathological conditions, a family of thioredoxin-mimetic (TXM) tri- and tetra-peptides was developed, based on the redox-active sequence motif of thioredoxin. These low-molecular-weight amino peptides each bearing two thiol groups, serve as a versatile platform of diverse redox-active molecules. Structurally optimized with blocked N- and C-termini, the TXM peptides exhibit enhanced cell permeability and are capable of crossing the blood–brain-barrier (BBB), thereby enabling therapeutic protection in both systemic degenerative and neurodegenerative disorders. Upon cellular entry, TXM peptides may undergo hydrolysis, potentially generating a cluster of 10–15 Cys-containing fragments including Cys, a precursor of GSH, which could further enhance and prolong their redox activity. This review summarizes key findings on the functional activity of various TXM-peptides, as demonstrated in both in vitro and in vivo models. Particular emphasis is given to TXM-CB3, which has shown protective effects across a wide range of animal models, including those of asthma, mild traumatic brain injury, obesity, viral infection, epilepsy, wound healing, myocardial infarction, aging, and inflammatory bowel-disease. These findings highlight the therapeutic potential of TXM-peptides in protecting cellular function under diverse pathological conditions.

## Abbreviations

AD4/NACA: N-acetylcysteine amide
AD7: *N*-acetyl glycine cysteine amide
AMD: age related macular degeneration
ASK1: Apoptosis signal-regulating kinase 1
AuF: auranofin
BBB: blood–brain barrier
CMs: cardiomyocytes
COPD: chronic obstructive pulmonary disease
CREB: cyclic AMP response element-binding protein
CVD: cardiovascular diseases
Cys: cysteine
ERK1/2: extracellular signal-regulated kinases 1 and 2
GSH: glutathione
JNK: c-Jun N-terminal protein kinase
LPS: Lipopolysaccharides
MAPK: mitogen activated protein kinase
MAP3K: mitogen-activated protein kinase kinase kinase
MI: myocardial infarction
mTBI: mild traumatic brain injury
NAC: N-acetylcysteine
NLRP3: nucleotide-binding oligomerization domain (NOD)-like receptor protein-3
NF-κB: Nuclear factor kappa-light-chain-enhancer of activated B cells
p38^MAPK^: p38 mitogen activated protein
ROS: reactive oxygen species
RNS: reactive nitrogen species
RPE: retinal pigment epithelia
Trx1: Thioredoxin1
TrxR: Thioredoxin reductase
TXM: Thioredoxin mimetic
TXNIP: Thioredoxin-interacting protein
VEGF: Vascular endothelial growth factor

## 1 Introduction

Thioredoxin1 and 2 are central components of the cellular redox system, playing a critical role in maintaining intracellular redox homeostasis and protecting both neuronal and non-neuronal cells from oxidative damage. This Trx redox system includes Trx1 within the cytosol and Trx2 within the mitochondria, Trx reductases (TrxRs), and NADPH. Electrons emanating from NADPH reduce intracellular disulfide using seleno-enzymes TrxRs. These enzymes reduce oxidized Trx (Trx-disulfide; Trx_ox_) at the active site yielding reduced Trx-dithiol (Trx_red_) and NADP^+^[1]. Similarly, glutathione-disulfide (GSSG)-reductases (Gsrs), reduce intracellular disulfide bond of GSSG using electrons from NADPH, yielding NADP^+^ and 2GSH. Both Trx_red_ and GSH further reduce selective substrates via dithiol-disulfide exchange reactions [2,3].

Trx1 is a highly conserved 12-kDa protein, whose functional impairment has been implicated in a wide range of neurological and non-neurological disorders, including Alzheimer's disease, Parkinson's disease, diabetes, chronic obstructive pulmonary disease (COPD), and age-related macular degeneration (AMD). Trx1 exerts its protective effects by catalyzing thiol-disulfide exchange reactions, reducing oxidized targeted proteins through two cysteine (Cys) residues within its redox-active motif, -Cys-Gly-Pro-Cys- [4]. The -CxxC- motif of Trx1, as well as the -CxC- motif, are conserved structural features shared among various redox enzymes, including Trx2 and glutaredoxin, underlining their importance in redox signaling and cellular defense mechanisms.

To strengthen endogenous defenses against redox imbalance and to protect neuronal cells from oxidative and inflammatory damage under pathological conditions, a series of low molecular weight thiol peptides was developed based on the redox motif of Trx1 peptides.

**Fig. 1:**
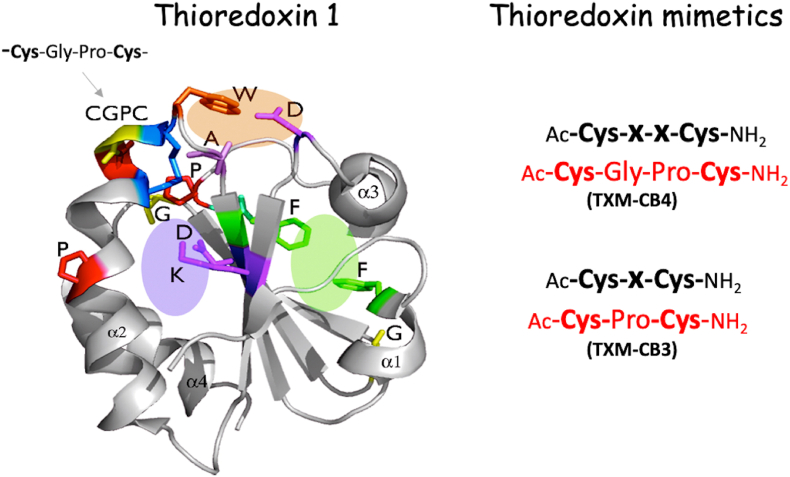
The structure of thioredoxin and its reactive redox motif, and thioredoxin mimetic peptides TXM-CB4 and TXM-CB3.

Building on the principle that derivatization of the carboxyl (COOH) group of the BBB-impermeable N-acetylcysteine (NAC) to an amide form yields the BBB-permeable NAC-amide (AD4/NACA), which significantly enhances redox activity and neuroprotective efficacy compared to NAC [5], the developed peptides were COOH- and NH_2_-blocked (Fig. 1). Designed to mimic the redox activity of Trx, these tri- and tetrapeptides, each containing two Cys-residues, incorporate the canonical redox-active motifs of the Trx1-active site sequence -Cys-X-X-Cys-as well as the shorter -Cys-X-Cys-motif characteristic of mitochondrial thiol oxidoreductases, were termed Trx mimetic (TXM) peptides [[6], [7], [8], [9]]. In the present review I will present a broad range of in vitro and in vivo studies evaluating the efficacy of these TXM peptides in protecting both neuronal and non-neuronal cells across diverse neurodegenerative and degenerative models, including Alzheimer's disease, Parkinson's disease, diabetes, chronic obstructive pulmonary disease (COPD), mild traumatic brain injury (mTBI), myocardial infarction, inflammatory bowel disease, and others.

## 2 In vitro studies: protective effects of TXM-peptides

### 2.1 Protection of primary neuronal cells from Aβ(1–42) toxicity

The beta-amyloid peptide (Aβ) deposits in the vascular tissue of Alzheimer disease (AD) brains revealed that the predominant 42-residue form Aβ(1–42) peptide is prone to aggregation into oligomers and fibrils. This peptide is considered the primary pathogenic species, whose toxicity has been associated with increased protein nitration and carbonylation, indicative of OS-induced damage in neuronal cells. Initial study by Bartov et al., was conducted in primary neuronal cultures, in which exposure to Aβ(1–42) led to an approximately 40 % reduction in cell viability [6]. Pretreatment with the TXM peptide Ac-Cys-Gly-Pro-Cys-NH_2_ (TXM-CB4) (Table I), almost completely reversed the cytotoxic effect at two fold higher concentrations as compared to smaller effects exhibited by single Cys antioxidants, AD4 (known as AD4/NACA), and AD7 (Ac-Cys-Gly-amide) [6,10]. TXM-CB4 also displayed superior neuroprotective potency in reversing the Aβ(1–42) induced oxidative damage as shown by reduced levels of protein carbonyls, 3-nitrotyrosine, and lipid peroxidation. In the same study the redox modulation activity of TXM-CB4, was further demonstrated by its ability to suppress the cisplatin- and the H_2_O_2_ -triggered activation of the mitogen activated protein kinase (MAPKs), p38 mitogen activated protein (p38^MAPK^) and extracellular signal-regulated kinases 1 and 2 (ERK1/2).

**Table 1:** List and structure of tri-and tetra-TXM-peptides.

Name	Cys (#)	Residue (#)	Peptide structure
TXM-CB3	2	3	Ac Cys Pro Cys NH_2_
TXM-CB4	2	4	Ac Cys Gly Pro Cys NH_2_
TXM-CB6	2	4	Ac Cys Gly Ala Cys NH_2_
TXM-CB8	2	3	Ac Cys Ile Cys NH_2_
TXM-CB10	2	3	Ac Cys DPro Cys NH_2_
TXM-CB13	2	4	Ac Cys Met Lys Cys NH_2_
TXM-CB15	2	3	Ac Cys γGlu Cys NH_2_
TXM-CB16	3	4	Ac Cys γGlu Cys Cys NH_2_
TXM-CB20	2	3	Ac Cys Gly Cys NH_2_
TXM-CB30	2	3	Ac DCys Gly DCys NH_2_
SD	2	3	Ac Cys l-DOPA Cys NH_2_

**Fig. 2:**
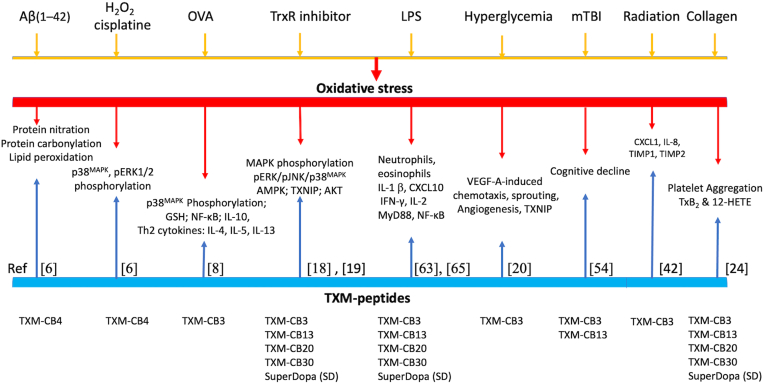
Antioxidant and anti-inflammatory roles of TXM-peptides in degenerative and neurodegenerative disorders.

These protective effects were attributed to the combined redox actions of the two Cys residues in TXM-CB4. Upon proteolysis, TXM-CB4 releases Cys residues that can serve as GSH precursor, chelate Cu^2+^ and Zn^2+^ ions, scavenge ROS such as superoxide radicals (O_2_^•-^) and reduce H_2_O_2_ to molecular oxygen and water. In this study, TXM was proposed as a potential therapeutic candidate for inhibiting Aβ(1–42)-induced neurotoxicity associated with Alzheimer's disease (Fig. 2; Table II).

**Table 2:** TXM peptides protect diverse intracellular signaling pathways: insights from in vivo and in vitro models.

Insult	In vitro	In vivo	Activity/Pathways	Peptide	Ref
Aβ(1−42)	Primary neuronal cells	–	Protecting against 3-nitrotyrosine, lipid peroxidation, & protein oxidation	TXM-CB4	[6]
Cisplatin (CDDP)	NIH3T3	–	Lowering pERK1/2 & p38^MAPK^ phosphorylation	TXM-CB4	[6]
H_2_O_2_	NIH3T3	–	Lowering pERK1/2 & p38^MAPK^ phosphorylation	TXM-CB4	[6]
Ovalbumin	NIH3T3	Mice	Enhancing IL-10 expression; attenuating NF-κB; Lowering Th2 cytokines: IL-4, IL-5, IL-13	TXM-CB3	[8]
Obesity (ZDF model)	INS-1832/13 cells	Brain of Zucker rats	Lowering MAPK activity Increasing insulin release; Preventing caspase 3 cleavage, PARP dissociation; Decreasing TXNIP/TBP-2; Increasing pAMPK	TXM-CB3	[18]
Diabetes type II and obesity model	HepG2cells	db/db mice (C57BLKS/J)	Lowering p38^MAPK^, ERK1/2, AKT, GSK3β & TSC2 activity in liver of db/db mice	TXM-CB3 TXM-CB13 TXM-CB16	[19]
mTBI		Mice (Male ICR)	Restoring Cognitive decline	TXM-CB3 TXM-CB13	[54]
TrxR inhibitor (auranofin)	Bovine chromaffin cells	–	Restoring Catecholamine release		
Hyperglycemia	HUVEC cells	–	Downregulation of VEGFR-2; Restoring survival migration, & proliferation	TXM-CB3	[20]
Collagen	Human Platelets	–	Reducing TxB_2_ & 12-HETE levels; Prolonging clotting time	TXM-CB3 TXM-CB13 TXM-CB30	[24]
Photic & non-photic stress	ARPE-19 cells	–	Protecting from rose Bengal (rB), rhodopsin-rich POS, sodium iodide; Reducing DPPH radical & singlet-oxygen quenching	TXM-CB3 TXM-CB13 TXM-CB30 SuperDopa	[36]
Radiation	HaCaT; HUVEC	–	Decreasing CXCL1, IL-8, TIMP1, TIMP2; improved cell survival	TXM-CB3	[42]
Chronic inflammation	macrophages Age-related Diseases	–	Preventing p21^CIP1^ upregulation; Maintaining proliferative activity	TXM-CB3	[44]
Acrylamide	PC12 cells	–	Lowering MAPK phosphorylation	TXM-CB3 TXM-CB13 TXM-CB16 TXM-CB20 TXM-CB30	[32]
LPS	murine macrophages	–	Lowering ROS levels of monocyte chemoattractant protein-1, IL-1 β, IL-6, (TNF)-α; Inhibiting NF-κB nuclear-translocation	TXM-CB3	[57]
High fat diet	–	ApoE2.Ki mice	Atheroprotective; Reducing plasma levels of IL-33, TNF-α, & OS markers; Lowering M1/M2 macrophage ratio; Reducing surface area of aortic lesions; Elevating adiponectin, IL-10	TXM-CB3	[57]
Myocardial infarction	Cardiomyocytes (CMs) macrophages	Neonate mice	Decreasing expression of cardiac inflammatory markers; Reducing apoptosis, OS; Increasing CM proliferation; Lowering macrophages M1/M2 ratio in spleen	TXM-CB3	[61]
SARS-CoV-2 & HIV infection	–	Mice	Inhibiting viral entry and replication of **SARS-CoV-2 varinets; Preventing spike protein binding to ACE2 receptor, syncytia formation;** Blocking entry of HIV-pseudo-typed viruses; Attenuating LPS-induced cytokine production	TXM-CB3 TXM-CB30 SuperDopa	[63]
Reductive stress	–	Mice expressing 667 GBM cells	Unique vulnerability of GBM to Cys-mediated redox imbalance -reductive stress in vivo	TXM-CB4	[64]
Colitis LPS-induced NLRP3 inflammasome	LPS-induced NLRP3 macrophages	DSS-induced colitis mouse model	Suppressing levels of NLRP3, Mlck, IL-1β in colonic tissues Upregulating Occludin, ZO-1 expression; Increasing NLRP1 expression; Inhibiting LPS-induced TLR4 signaling; Reducing MyD88 expression & NLRP3, ASC, Caspase-1, Gasdermin D, IL-1β	TXM-CB13	[65]
Rotenone	SH-SY5Y cells-	Mice	Relief of motor disabilities; Lowering auranofin-induced JNK1, & p38^MAPK^phosphorylation	SuperDopa (SD)	[70]

### 2.2 Restoration of oxidative stress impaired catecholamines release

Depolarization-evoked neurotransmitter release is highly sensitive to oxidative conditions, mainly due to the oxidation of Cys271 and Cys272 within the transmembrane domain of syntaxin 1A [11]. Treatment of bovine chromaffin cells with auranofin (AuF), a selective thioredoxin reductase (TrxR) inhibitor, resulted in a complete inhibition of catecholamine release, as demonstrated by amperometry recordings in single chromaffin cells [7] (Table II). This AuF-induced inhibition of neurotransmitter release was fully reversed in a dose-dependent manner by the TXM-CB4, AcCys-Gly-Ala-CysNH_2_ (TXM-CB6), and AcCys-Pro-CysNH_2_ (TXM-CB3) [7]. In the same study these TXM peptides also effectively reversed AuF-induced MAPK activation in PC12 cells. Their redox modulating efficacy was significantly greater than that of conventional single-Cys antioxidants including NAC, GSH, DTT, and NAC-amide (AD4/NACA). The reversible redox sensitivity of the exocytotic machinery as shown in these studies was further confirmed using bovine chromaffin cells [12], and highlights the superior reducing potency of the TXM family, likely mediated via the apoptosis signal-regulating kinase 1 (ASK1), which is member of the mitogen-activated protein kinase-kinase-kinase (MAP3K) family [7,9,12,13].

### 2.3 Protection of insulinoma cells from apoptosis and restoration of impaired insulin release

Disruption of the TrxR/Trx1 system by AuF, a highly potent TrxR inhibitor, induces Trx1 oxidation, leading to the activation of the ASK1/MAPK apoptotic pathway and subsequently to cell death. The protective effects of TXM peptides against AuF-induced apoptosis were evaluated in the rat insulinoma INS-1832/13 cell line, which contains a human insulin expression cassette enabling secretion of human insulin [9] (Fig. 2; Table II). In this cellular model, Cohen et al., have shown that both TXM-CB3 and TXM-CB4 effectively reversed the AuF-induced activation of the ASK1/MAPK apoptotic signaling cascade. The inhibition of Auf-induced MAPKs, c-Jun N-terminal protein kinase (JNK) and p38^MAPK^ phosphorylation, was correlated with the prevention of caspase-3 cleavage and subsequent poly (ADP-ribose) polymerase-1 (PARP-1) dissociation. These results indicate the capacity of TXM-peptides to restore redox homeostasis, lower inflammatory activity, and to support cell survival under OS conditions [9].

Most interestingly, in these cells membrane depolarization triggers insulin release, a process that is highly redox sensitive. To investigate the mechanisms underlying insulin secretion, TXM-CB3, TXM-CB4, and AcCys-DPro-CysNH_2_ (TXM-CB10) (Table I), were tested and shown to restore AuF-impaired insulin secretion. The effect attributed to the oxidation of vicinal Cys residue in Syntaxin 1A, highlights the ability of TXM peptides to rescue catecholamine release in bovine chromaffin cells, further underscoring their therapeutic potential in counteracting OS-induced dysfunction of vesicular exocytosis [7,12] (see Section 2.2). These findings suggest a broader therapeutic potential of TXM peptides in preserving secretory functions across different neuroendocrine systems affected by redox imbalance. This activity may become valuable in the treatment of OS related disorders such diabetes (see also section 2.5) on TXM-CB3 effect on hyperglycemia-induced endothelial dysfunction, Section 3.2 for TXM-CB3 effect on obese Zucker rats and Section 3.3 for TXM-CB3 effect on db/db mice.

### 2.4 Catalysts of S-denitrosylation and anti-nitrosative agents

The thioredoxin-reductase/thioredoxin (TrxR/Trx) system functions analogously to the glutaredoxin and lipoic acid systems, maintains cellular redox homeostasis by preserving substrates in a reduced free-thiol state under nitrosative stress conditions. Excessive NO causes aberrant S-nitrosylation reactions disrupting protein and mitochondrial function, contributing to the pathogenesis of neurodegenerative diseases such as Alzheimer's and Parkinson's diseases [14]. The protective function of Trx1 is primarily mediated through the **S-denitrosylation** of S-nitrosylated protein substrates/inflammatory [15,16]. To further investigate the functional similarity of TXM-peptides to the well characterized denitrosylating activity of Trx1, their ability to catalyze denitrosylation was tested both in a cell-free system and in cultured cells subjected to nitrosative stress [17]. This biochemical analyses revealed that both TXM-CB3 and TXM-CB4 exhibit an S-nitrosothiol (SNO) reductase activity, effectively catalyzing the denitrosylation of low-molecular weight SNO compounds, such as S-nitrosoglutathione (GSNO) [9,17]. Moreover, both TXM-CB3 and TXM-CB4 appeared to synergize with an NADPH-dependent activity to facilitate protein denitrosylation. TXM-CB3 enhanced GSNO reduction through functional coupling with TrxR. These findings suggest that beyond their established antioxidant properties, **TXM-peptides also possess denitrosylating activity**, consistent with their functional similarity to the endogenous TrxR/Trx1 system.

### 2.5 Restoration of hyperglycemia-induced endothelial dysfunction through downregulation of VEGFR-2

Earlier studies demonstrated reversal of inflammatory apoptotic markers in Zucker rats predisposed to obesity by TXM-CB3 [18] (see Section 3.2), and inhibition of inflammatory pathways in the liver of db/db mice [19] (see Section 3.3). These findings were further extended by Hemling et al., who investigated the potential of TXM-CB3 to reduce cardiovascular risk factors associated with diabetes mellitus [20]. They explored impaired Trx activity resulting from elevated plasma glucose levels linked to hyperglycemia, which are known to adversely affect endothelial cell function, and diminish responsiveness to vascular endothelial growth factor-A (VEGF-A). In this study, human umbilical vein endothelial cells (HUVECs) exposed to hyperglycemic conditions using the glucose metabolite methylglyoxal displayed diminished chemotactic responses to VEGF-A stimulation, indicating the development of a VEGF-resistant phenotype (Fig. 2; Table II). These HUVECs exhibited impaired migratory and proliferative responses, along with a shift toward a pro-apoptotic state, attributed to elevated ROS levels resulting from compromised Trx1 activity [20]. Treatment with TXM-CB3 under these high-glucose conditions effectively reduced ROS levels using CellROX reagent, improved cell migration, proliferation, and survival, and restored VEGF-A-induced chemotaxis and sprouting angiogenesis. Furthermore, TXM-CB3 treatment reduced ROS accumulation and improved VEGF-A responsiveness in placental arterial endothelial cells isolated from gestational diabetes mellitus patients. These findings led to suggest that TXM-CB3 may represent a novel therapeutic strategy for restoring endothelial cell function in diabetes.

### 2.6 Inhibition of platelet aggregation

Platelet activation during oxidative metabolism is closely linked to the mobilization of arachidonic acid from membrane phospholipids, primarily mediated by phospholipase A_2_ (PLA_2_). Once released, arachidonic acid undergoes enzymatic conversion via the cyclooxygenase (COX) pathway, leading to the formation of bioactive eicosanoids such as thromboxane A_2_ (TXA_2_) and 12-hydroxyeicosatetraenoic acid (12-HETE). Both TXA_2_ and 12-HETE play key roles in promoting platelet adhesion, aggregation, and the amplification of thrombotic responses, particularly under conditions of oxidative stress [21,22]. In earlier studies, efforts to prevent thrombus formation demonstrated that treatment with NAC inhibited the release of TXA_2_ and 12-HETE in macrophages [23]. These findings prompted further investigation of TXM peptides, including TXM-CB3, TXM-CB30, and TXM-CB13 [24]. Both TXM-CB3 and TXM-CB30 significantly reduced TxB_2_ and 12-HETE levels, and prolonged the clotting time of whole blood, indicating their potential antithrombotic effects. In addition, TXM-CB3 increased the levels of antioxidants free thiol groups in plasma. By restoring redox balance and interfering with platelet aggregation pathways, TXM peptides could play a critical role in preventing thrombus formation, a key pathogenic mechanism in acute coronary syndromes, including unstable angina and acute myocardial infarction **(see** section 3.7**; and** (Fig. 2; Table II)**). These results** suggest a therapeutic potential of TXM-peptides in conditions driven by inflammation and OS involving platelet activation.

### 2.7 TXM-CB3 rescues H_2_O_2_-induced decrease in CREB activation in primary mouse cortical neuronal cell culture

Upregulation of Trx has been suggested to support neuronal survival and differentiation by enhancing CREB phosphorylation [[25], [26], [27]]. To test whether suppression of inflammatory signaling confers neuroprotection by modulating redox pathways [[28], [29], [30]], the role of CREB in mediating Trx-dependent neuronal survival and differentiation was examined. In this study, knockdown of Trx expression in cultured neurons using Trx sgRNA CRISPR/Cas9 technology resulted in reduced Trx levels, which were accompanied by decreased dendritic outgrowth and branching of cortical neurons. This structural reduction correlated with diminished CREB activation, suggesting a critical role for Trx in neuronal differentiation [31]. Furthermore, oxidative stress induced by H_2_O_2_ lowered CREB activation in SH-SY5Y cells, an effect that was reversed by TXM-CB3 treatment. These findings are consistent with previous studies demonstrating the ability of TXM peptides to protect the ERK/CREB pathway from oxidative stress [[6], [7], [8], [9],^32^].

### 2.8 Protection of retinal ARPE-19 cells from photic- and non-photic stress

There is a well-established correlation between OS and the pathogenesis of retinal diseases, including diabetes retinopathy, age-related-macular-degeneration (AMD), retinitis pigmentosa and others [33]. Elevated levels of ROS in the retina have been associated with morphological abnormalities and dysfunction of the retinal pigment epithelia (RPE) cells. This single postmitotic cell layer plays a key role in the metabolic support of photoreceptor cells and in protecting them from age-related degeneration and light-induced damage. Antioxidant supplementation has shown some efficacy in delaying disease progression, prompting the investigation of the protective properties of TXM family members. To evaluate the protective effects of TXM-peptides against photic stress, we employed Rose Bengal, a xanthene family dye that functions as a photosensitizer upon irradiation with green light (520–580 nm). When activated, Rose Bengal generates reactive singlet oxygen (^1^O_2_), along with additional ROS, leading to oxidative damage. Photic stress was further evaluated using rhodopsin-rich photoreceptor outer segments (POS) exposed to green light, which increases the risk of phototoxic reactions mediated by rhodopsin bleaching products.

Protection against non-photic stress was assessed with sodium iodate, a widely used oxidizing agent that induces retinal degeneration by triggering the death of retinal pigment epithelium (RPE) cells [34,35].

Using ARPE-19 cells, a spontaneously derived RPE cell line, Olchawa et al., [36], demonstrated that TXM-CB13, TXM-CB30, and SuperDopa (SD) (see Section 4.2) effectively protected against both photic stress-induced by Rose Bengal or rhodopsin-rich POS irradiated with green light, which reduces cell survival, and non-photic stress caused by sodium iodate-induced oxidation [36] (Table II).

Protection from OS s correlated with a reduction in DPPH radical activity and singlet-oxygen quenching. These retino-protective peptides have been proposed as potential drug candidates for moderating RPE dysfunction, destined for slowing the onset and progression of RPE associated disorders like retinitis pigmentosa, diabetic retinopathy, or AMD.

### 2.9 Regeneration of mercaptoalbumin, the reduced form of human serum albumin

Human-serum-albumin (HSA) constitutes approximately 50 % of the total plasma protein in healthy individuals and contributes significantly to the plasma's overall antioxidant and pro-oxidant buffering capacity. The break-down of mixed disulfides of albumin (HSA) yields the reduced form mercaptoalbumin (HMA), and the oxidized form, whose predominant modification is cystenylation (HSA-Cys) [37]. The anti-inflammatory, anticoagulant, and anti-aggregating properties of HSA-Cys are primarily attributed to its redox-active **Cys34** residue, making it a valuable biomarker in pathological conditions [38]. Partial restoration of the inhibitory effect on platelet aggregation by NAC [37,39], led Eligini et al., to examine the efficacy of TXM-peptides in regenerating the reduced-proteoform of HSA (HSA-SH) [40]. In this study TXM-CB3, TXM-CB13, and TXM-CB30 were tested alongside the single Cys compound AD4/NACA, in regenerating the reduced-proteoform of HSA using plasma samples obtained from three healthy subjects. The relative abundance of HSA-SH was detected by LC-MS and visualized with dichlorofluorescin fluorescence [40]. Among the tested compounds, TXM-CB3 exhibited the highest potency, significantly reducing HSA-Cys levels already at 0.05 mM (−8.9 ± 7.56 %) and reaching a maximal effect at 0.6 mM (−81.24 ± 0.38 %) (Table II). Additionally, all compounds induced an increase in the number of free sulfhydryl groups in HSA, detectable within 5 min of incubation and remaining significantly elevated for up to 180 min. TXM-CB3 was the most potent, inducing approximate 530 % increase in HSA-SH levels after 10 min. These findings highlight an additional therapeutic advantage of TXM-peptides, indicating their ability to restore the reduced form of HSA in plasma and effectively lower levels of its oxidized proteoform.

### 2.10 Protection from radiation damages and improving wound healing

Although radiation is a key player in cancer therapy [41], it is associated with long-term adverse effects, including impaired tissue regeneration. These effects are primarily driven by radiation-induced ionization, which leads to excessive generation of ROS including superoxide anions, hydrogen peroxide, hydroxyl radicals, singlet oxygen, and peroxyl radicals.

In a study by Borrmann et al., the radioprotective effects of TXM-CB3 in particular those affecting the skin, were demonstrated utilizing cultured human epidermal keratinocytes (HaCaT) and primary human umbilical vein endothelial cells (HUVECs) exposed to 2 Gy ionizing irradiation [42]. TXM-CB3 significantly reduced radiation-induced ROS levels and DNA double-strand breaks in both HaCaT cells and HUVEC cultures. Cytokine array analysis revealed that pre-treatment with TXM-CB3 (24 h prior to irradiation) led to decreased expression of proinflammatory and tissue remodeling factors, including chemokine ligand 1 (CXCL1), IL-8, TIMP1, and TIMP2 (Fig. 2; Table II). Furthermore, cells pretreated with TXM-CB3 exhibited a significantly higher baseline cell survival rate and maintained a normal cellular phenotype across a range of radiation doses.

A wound healing test in the form of a scratch assay by pretreatment of TXM-CB3, as well as time-dependent migration and proliferation measurements via digital holographic microscopy with HaCaT, showed improved ability of the cells to close the gaps in the cell layer following 2Gy radiation (see also [20]). The radioprotective effects of TXM-CB3 were most effective when administered within a short window prior to radiotherapy treatment, suggesting its potential use as a therapeutic antioxidant for transdermal application of antioxidants for lowering radiation-induced tissue toxicity.

### 2.11 Managing chronic inflammation in models of age-related diseases (ARD)

The accumulation of senescent cells, marked by the senescence-associated secretory phenotype (SASP), plays a key role in driving chronic inflammation and age-related diseases (ARDs) [43]. With aging, macrophages can adopt a senescent-like phenotype, exhibiting altered immune functions that further promote the persistence and accumulation of senescent cells. A recent study by Smith et al., established an in vitro model of macrophage aging that exhibits a senescence-like phenotype associated with inflammatory, metabolic and functional alteration, using cultured murine peritoneal macrophages [44].

In a 14-day culture assay, macrophages exhibited reduced proliferative capacity, accompanied by a progressive increase in both mRNA and protein expression of the cyclin-dependent kinase inhibitors p16^INK4A^ and p21^CIP1^, as well as by an increase in the pro-inflammatory markers MCP-1, IL-6, IL-1β, TNF-α, and MMP-9 [44] (Table II). Additionally, these cells displayed enhanced glycolytic activity and a marked decline in phagocytic function over time. Using this experimental model, the study demonstrated that TXM-CB3 effectively prevented the development of a senescence-like phenotype. Specifically, TXM-CB3 completely prevented the upregulation of p21^CIP1^ throughout the 14-day period, and preserved macrophage proliferative activity, without significantly altering the expression levels of inflammatory markers. These results further support a protective role for TXM-CB3 in controlling chronic inflammation in aging-related pathologies, consistent with its antioxidant/anti-inflammatory activity.

### 2.12 Inhibition of acrylamide induced-MAPK activation

**Fig. 3:**
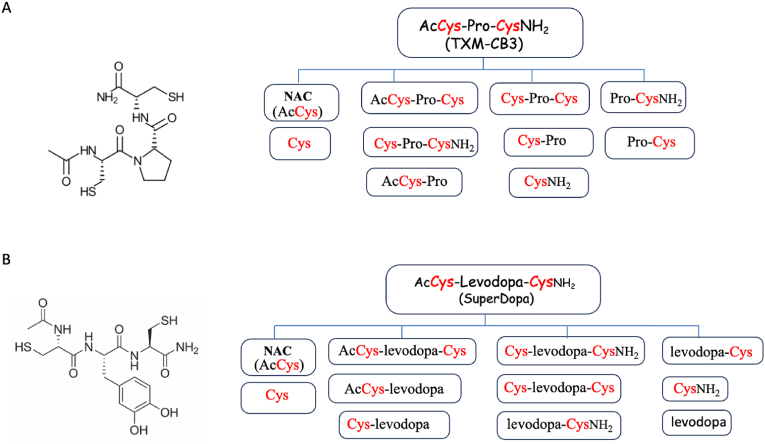
**The structure and proteolytic putative cleavage of TXM-CB3 and SuperDopa to generate a “Redox cluster” of Cys containing fragments including Cys and NAC** (**A**) Structure of TXM-CB3, an N- and C-blocked tri-peptide susceptible to proteolysis (*left*). Proteolytic cleavage may generate a cluster of up to 10 putative Cys-containing fragments including Cys and NAC (*right*) (**B**) Structure of SuperDopa, an N- and C-blocked tri-peptide susceptible to proteolysis (*left).* Proteolytic cleavage may yield up to 10 Cys-containing fragments including Cys and NAC, and up to 8 levodopa-containing fragments including levodopa (*right*).

Acrylamide (ACR) is a non-aromatic, low-molecular-weight compound widely used in industrial processes such as paper manufacturing, textiles, plastics, cosmetics, and dyes, which is formed during high-temperature cooking of starchy foods [45,46]. ACR toxicity primarily stems from its ability to covalently bind to thiol groups, particularly of GSH and Trx1, thereby disrupting the functionality of these critical redox systems. Martin et al., 2024., evaluated the efficacy of TXM-peptides in reversing acute ACR toxicity using the rat pheochromocytoma (PC12) cells as an in vitro model [32]. In this study, the reversal of ACR-induced toxicity was tested by monitoring MAPK activation using several members of the TXM peptide family, including the tripeptides AcCys-Gly-CysNH_2_ (TXM-CB20), AcDCys-Gly-DCysNH_2_ (TXM-CB30), and AcCys-Levodopa-CysNH_2_ (SuperDopa (SD) see Section 4; Fig. 3B), the tetrapeptides TXM-CB13, and the 3-Cys tetrapeptide, AcCys-γGlu-Cys-CysNH_2_ (TXM-CB16) (Table I). All tested peptides effectively inhibited ACR-induced activation of both the ERK1/2 and the p38^MAPK^ apoptotic/inflammatory pathways (Table II). TXM-peptides containing two or three Cys residues, demonstrated significantly greater efficacy compared to the single-Cys antioxidants such as NAC and AD4/NACA. These results further support the superior protective potential of multi-Cys TXM-peptides and their wider applicability in reversing a wide range of OS related pathologies.

## 3 In vivo studies: protective effects of TXM-peptides

### 3.1 TXM-CB3 protects against pulmonary obstruction in an OVA-induced asthma model

ROS have been shown to play a critical role in the pathogenesis of airway inflammation and tissue injury in asthma, contributing to epithelial cell damage, cell shedding, and airway hyperresponsiveness [47,48]. Involvement of ROS in airway disease was further confirmed by impaired endogenous redox defense system in asthma patients. The pathophysiological features of asthma were significantly attenuated by both NAC and AD4/NACA in vivo, as demonstrated in the ovalbumin (OVA)-induced murine model of asthma [49]. These studies led Kim et al., examined the effects of TXM-CB3 using the same OVA-inhaled murine model [8]. Administration of TXM-CB3 (1–50 mg/kg) to OVA-inhaled mice restored GSH levels, enhanced IL-10 expression, and significantly attenuated the increase of Th2 cytokines (IL-4, IL-5, IL-13) in lung tissues, bronchoalveolar lavage (BAL) fluids, and serum (Fig. 2; Table II). In these studies TXM-CB3 also showed a significant reduction in inflammatory cell infiltration and airway hyperresponsiveness in the lungs, accompanied by a decrease in nuclear translocation of the nuclear factor-κB (NF-κB) and reduced phosphorylation of p38^MAPK^ in the OVA-inhaled lungs [8]. Consistent with the central role of inflammation and OS in allergic airway disease, these findings demonstrated for the first time the in vivo efficacy of TXM-CB3. They revealed that its therapeutic potential is most likely driven by the anti-inflammatory and antioxidant activities, with significantly greater potency compared to AD4/NACA. These results highlight TXM-CB3 as a promising candidate for the treatment of airway associated disorders.

### 3.2 Attenuation of neuroinflammatory processes in the brain of obese (zucker) rats

The pathological impact of obesity on the central nervous system (CNS) has been linked to impaired ability of insulin resistance, caused by the inability of insulin to act on its target receptors, and to chronic low-grade peripheral inflammation, both of which may contribute to the development of neurodegenerative diseases, including cognitive dysfunction and dementia [50,51].

Cohen-Kutner et al., [18] investigated the contribution of TXM-peptides to alleviating inflammation- and reversal of OS-activated MAPK pathways associated with obesity in the brain. They monitored MAPKs phosphorylation, AMP-ribose activating kinase (AMPK) phosphorylation, expression of thioredoxin-interacting-protein (TXNIP/TBP-2), and the inhibition of p70(S6K) kinase in the brains of male leptin-receptor-deficient Zucker diabetic fatty (ZDF) rats. Six-weeks old male Zucker rats were evaluated following i.p injection with either TXM-CB3 (1 mg/kg and 10 mg/kg) or with rosiglitazone, for 28 days. Analysis of brain samples revealed a significant reduction in the phosphorylation of JNK and p38^MAPK^, along with decreased expression of TXNIP/TBP-2 (Fig. 2; Table II; see Section 3.9). These changes were correlated with lower levels of the apoptotic markers, caspase 3 and cleaved PARP, as observed in SH-SY5Ycells. Most significant were the TXM-CB3-induced increase in the phosphorylation of AMPK, and the decrease in the phosphorylation of p70S6K, likely mediated through modulation of MAPK-AMPK-mTOR signaling pathway. The TXM-CB3 protective effects resembled those elicited by rosiglitazone, a drug known for increasing the body's sensitivity to insulin. The findings in the ZDF rat model of obesity/type 2 diabetes suggest that TXM-CB3 through its attenuation of neuroinflammatory processes, may hold therapeutic potential for preventing neurological disorders associated with high-glucose diets and obesity.

### 3.3 TXM-CB3 treatment effectively inhibits inflammatory signaling pathways linked to high glucose levels in db/db mice

In diabetes, prolonged exposure to high glucose levels is associated with chronic inflammation, which drives disease progression [52] (see Section 3.2). This inflammatory state in diabetic patients correlates strongly with elevated expression of OS markers. Chronic inflammation also leads to severe alterations in insulin signaling and extensive protein post-translational modification, which are considered hallmarks of both chronic metabolic disorders and neurodegenerative diseases. In a study by Lejnev et al., [19] the protective effects of TXM-CB3 were further demonstrated in the liver of db/db mice, a well-established murine model of severe obesity, glucose intolerance, sustained hyperglycemia, and dyslipidemia, caused by an inactivating mutation in the leptin receptor gene. This model is widely used to characterize the metabolic and histological effects of anti-diabetic drugs in various phases of type 2 diabetes. Beginning at 6 weeks of age, db/db mice received daily i.p injections of either TXM-CB3 (5 mL/kg) or Rosiglitazone for 21 days. Intraperitoneal administration of TXM-CB3 significantly lowered the elevated activities of MAPKs, including phospho-ERK1/2 and phospho-p38^MAPK^, as well as phospho Akt/PKB observed in the liver of db/db mice (Fig. 2; Table II**)**. These results are consistent with the anti-inflammatory impact of TXM-peptides on MAPKs that have been demonstrated both in vivo, in the brain of obese Zucker rats [18] (see Section 3.2), and in vitro, where TXM peptides restored endothelial cell function under high-glucose conditions [20] (see Section 2.5). Treatment with rosiglitazone, an antidiabetic drug, displayed comparable reductions in activated ERK1/2, p38^MAPK^, and Akt/PKB. TXM-CB3 also reversed in insulin- and OS-induced phosphorylation of ERK1/2 and AKT/PKB in FAO hepatoma cells, further supporting its role in modulating OS-induced signaling pathways associated with hyperglycemia and metabolic disfunction.

### 3.4 TXM peptides preserve cognitive function following mild traumatic brain injury (mTBI)

Mild traumatic brain injury (mTBI) and spinal cord injuries are among the most common battlefield injuries in soldiers, as well as in children, athletes, and the elderly. Despite their prevalence, therapeutic options remain limited. mTBI is often associated with long-lasting cognitive, behavioral, and emotional difficulties. NAC treatment has shown protective effects in U.S service members deployed to Iraq who experienced mTBI, suggesting that OS and inflammation play a critical role in injury outcomes [53]. This finding led to testing TXM-CB3, and TXM-CB13 (Table I) for their ability to provide cognitive protection following mTBI [54]. A single intraperitoneal dose of TXM-CB3 or TXM-CB13 (50 mg/kg) administered 1h post injury significantly improved cognitive function in a weight-drop closed-head injury mouse model. Cognitive performance was evaluated at 7 and 30 days post injury, using two independent behavioral assays, the Y-maze and Novel Object Recognition (NOR) test. Both peptides demonstrated substantial neuroprotective effects, indicating their potential as therapeutic agents for mitigating mTBI-induced cognitive deficits (Fig. 2; Table II). In this study, TXM-CB13 similar to TXM-CB3 and AD4/NACA, effectively reversed OS-induced phosphorylation of p38^MAPK^ and JNK in human neuronal SH-SY5Y cells. Both in vivo and in vitro results demonstrated the superior potency of the tetrapeptide TXM-CB13 compared to AD4/NACA (Section 2.12; [32]). These findings highlight the therapeutic potential of a single post-injury administration of TXM-peptides, suggesting their promise in preventing long-term damage following brain trauma such as the development of chronic encephalopathy observed in American football players and others exposed severe head injuries. The sustained behavioral effects may be attributed to the cluster effect of TXM-CB3 (Fig. 3A, *right*), which upon proteolysis can generate up to 10 distinct Cys-containing proteolytic fragments [10]. Although Cys cannot substitute for GSH for protecting cells against OS, it may serve as a GSH precursor that prevents GSH depletion.

### 3.5 Athero-protective effects of TXM-CB3 in ApoE2.Ki mice fed a high-fat diet

Cardiovascular diseases (CVD), largely driven by chronic low-grade inflammation of the arterial wall, remain the leading cause of morbidity and mortality worldwide. The sustained inflammatory process contributes to atherosclerotic complications promoting monocytes infiltration into vascular tissue and their subsequent differentiation into tissue-resident macrophages within the atherosclerotic lesions [55].

It has been shown that OS resulting from elevated ROS production in the vascular wall, triggers endothelium damage, and oxidation of low-density lipoproteins. Trx1 exhibits protection in CVD primarily by promoting macrophages polarization toward the anti-inflammatory M2 phenotype and by inhibiting lipopolysaccharide (LPS)-induced polarization toward the pro-inflammatory M1 phenotype [56]. The involvement of ROS and the Trx1 protective effects in CVD led Canesi et al., to examine the anti-inflammatory effects of TXM-CB3. They examined its effects in cultured peritoneal murine macrophages and in the ApoE2.Ki mice, a model that develops atherosclerosis when fed high-fat diet [57]. In these studies, TXM-CB3 significantly and dose-dependently reduced ROS levels and CB3 reduces ROS levels and H_2_O_2_ production in LPS-activated macrophages and attenuated LPS-induced inflammatory process by inhibiting NF-κB nuclear translocation (Table II). It also decreased the secretion of key pro-inflammatory cytokines and chemokines, including monocyte chemoattractant protein-1, interleukin-1β (IL-1 β), IL-6, and tumor necrosis factor (TNF)-α by macrophages. Daily i.p injection of TXM-CB3 (10 μg/g for 10 weeks) to ApoE2.Ki mice fed on high-fat diet, significantly reduced plasma levels of pro-inflammatory cytokines IL-33 and TNF-α, as well as OS markers, without affecting total cholesterol or triglycerides levels. TXM-CB3 also elevated the level of the anti-inflammatory proteins adiponectin, IL-10, decreased the number of pro-inflammatory M1 macrophages in spleen, lowered the M1/M2 macrophage ratio within atherosclerotic lesions, and reduced the surface area of aortic lesions. These athero-protective effects highlight TXM-CB3 treatment were suggested as a promising therapeutic candidate for the treatment of atherosclerosis (see Section 3.6 and Section 3.7).

### 3.6 TXM-CB3 improves systolic cardiac function and attenuates remodeling following myocardial infarction

Myocardial infarction (MI), a leading cause of morbidity and mortality worldwide [58], is characterized by a substantial loss of cardiomyocytes (CMs), primarily driven by elevated levels of ROS. In the mammalian neonatal heart a regenerative capacity to 7 postnatal days [59]. During exposure to an oxygen rich environment excessive ROS contributes to CM cell cycle arrest and impair their regenerative capacity, which During hypoxia and in the presence of ROS scavengers, the cell cycle can restart [60]. The resulting irreversible cellular damage and CM death during MI lead to coronary artery occlusion, ultimately compromising cardiac function by reducing blood flow and oxygen delivery to the myocardium. The cardioprotective effects of TXM-CB3 in MI were evaluated by Medali et al., in comparison to Adeno-Associated-Viruses of Trx1 (AAV-Trx1) and Trx-80 (AAV-Trx-80) [61]. In this study an experimental mice model of MI was established by permanent ligation of the left anterior descending coronary artery. Mice were treated with AAV-Trx1 or AAV-Trx-80 for one month prior to MI induction, or daily administration with TXM-CB3 (10 mg/kg). TXM-CB3 significantly improved cardiac function, as monitored by echocardiography, significantly increased GSH levels and resulted in a significant reduction in infarct size and myocardial fibrosis (Table II). These histological improvements were accompanied by decreased expression of inflammatory markers in the hearts of mice 3 weeks after experimental MI, and were correlated with improved cardiac performance, comparable to the improvement observed in 8-week-old mice pretreated with AAV-Trx1 but not with AAV-Trx-80 [61]. Compared to control mice, treatment of infarcted mice with CB3 significantly reduced the percentage of carbonylated protein. In neonate mice, TXM-CB3 protected cardiomyocyte from ROS-induced metabolic alterations, inflammation, apoptosis, and fibrosis, observed during a transient regenerative window spanning the first seven postnatal days [59,61].

Furthermore, in this experimental MI setup, TXM-CB3 shifted macrophages from the pro-inflammatory M1 into the anti-inflammatory M2 phenotype. This shift was associated with reduced OS-induced apoptosis, decreased mRNA expression of the cyclin-dependent kinase inhibitors p16^INK4A^ and p21^CIP1^, and enhanced CM proliferation. Similar immunomodulatory effects were observed in ApoE2.Ki mice, where TXM-CB3 significantly decreased the number of M1 macrophages in the spleen, reduced the M1/M2 macrophages ratio within atherosclerotic lesion areas, and diminished the surface area of aortic lesions [57] (see Section 3.5).

### 3.7 Inhibition of redox-sensitive SARS-CoV-2 and HIV infection by TXM peptides

Despite the success of vaccines and anti-viral drugs against SARS-COV-2, the search for effective therapeutics, particularly for long COVID-19 continues. The receptor binding domain (RBD) of the SARS-CoV-2 spike protein, which mediates viral entry through binding to the human ACE2 receptor, continues to serve as critical therapeutic target. Cell fusion is influenced by redox potential, primarily though alterations in the thiol-disulfide equilibrium within the RBD [62]. This redox-sensitive viral fusion is not unique for SARS-Cov-2 but is also a characteristic feature of several other viral-fusion proteins, including those of HIV, murine leukemia virus, human T-cell lymphotropic virus, rubella virus, sindbis virus, mouse-hepatitis virus, and bovine viral diarrhea virus. Given the essential role of thiol-disulfide exchange in the interaction between the SARS-CoV-2 spike protein RBD and ACE2 receptor, we examined the efficacy of several TXM-peptides in inhibiting syncytia formation, viral entry into cells, and infection in a murine model [63].

ln vivo, mice were i.p injected with TXM-CB3, TXM-CB30, or SD (Fig. 2; Table II) for two consecutive days (2 mg/mouse/day), followed by intranasal transfection with DNA plasmids encoding the human ACE2 receptor and TMPRSS2 [63]. One day later, the mice were infected with spike protein-pseudo-typed SARS-CoV-2 expressing firefly luciferase. Bioluminescence imaging revealed a significant reduction in viral entry, indicating strong inhibition of viral infection by all three TXM-peptides. Similarly, these peptides effectively blocked the entry of HIV-pseudo-typed viruses, another example of a redox-sensitive virus.

**Furthermore, TXM-CB3 inhibits viral entry and replication of several SARS-CoV-2 variants, including** the Wuhan, Alpha, Delta, and Omicron, after preincubation for 1h with TXM-CB3. Additionally, TXM peptides were shown to reduce the activation of NF-κB and the interferon regulatory signaling pathways, inhibit mitogen-activated MAPKs, and attenuate LPS-induced cytokine production in mice. Collectively, these findings support the potential of TXM peptides as a broad-spectrum antiviral strategy. They suggest that TXM peptides may offer a promising clinical approach for mitigating viral infections and reducing the severe inflammatory and OS consequences of COVID-19 and other redox-sensitive viruses including HIV, murine leukemia virus, human T-cell lymphotropic virus, rubella virus, Sindbis virus, and bovine viral diarrhea virus.

### 3.8 Reductive stress in glioblastoma through hydrogen peroxide production

Glucose and amino acid metabolism are essential for the growth of many cancers, including glioblastoma (GBM), which displays the highest expression levels of Cys- and methionine-metabolic genes among 32 human cancer types. Cys-containing compounds such as NAC have been shown to induce GBM cell death by disrupting mitochondrial function and driving bioenergetic failure primarily through reducing mitochondrial oxygen consumption and membrane potential. These findings led Noch et al., to examine the efficacy of the dithiol containing peptide TXM-CB4 in eliciting similar cytotoxic effects to those observed with NAC [64]. Initial in vitro studies using 667 glioblastoma cells demonstrated that TXM-CB4 was 100–200 fold more potent than NAC in reducing oxygen-consumption rate, indicating a pronounced inhibitory effect on mitochondrial respiration. These findings are consistent with the electron acceptor function of Cys93 and Cys95 residues in Trx2, whose mutation to Ser, abolishes Trx2-mediated rescue of H_2_O_2_ production. The ability of TXM-CB4 to increase the level of ROS and H_2_O_2_ production in an orthotopic GBM mouse model, was tested in vivo using luciferase-expressing 667 GBM cells. The cells were implanted intracranially into NSG mice and allowed to form tumors until a predefined bioluminescence threshold was reached. Following tumor establishment mice were treated i.p with either vehicle, 2-deoxyglucose (2-DG), or a combination of 2-DG and TXM-CB4. Treatments were administered twice daily, five days per week. Combined treatment with 2-DG and TXM-CB4 resulted in a significantly higher bioluminescent signal for H_2_O_2_ in orthotopic xenografts compared to treatment with 2-DG or vehicle alone, indicating enhanced OS within the tumor microenvironment (Table II) [64]. These findings demonstrate that TXM-CB4 treatment induces reductive stress in vivo and further confirm the unique vulnerability of GBM to Cys-mediated redox imbalance. This vulnerability exacerbated by glucose deprivation, warrants further investigation as a potential therapeutic strategy for GBM.

### 3.9 Amelioration of OS-mediated adverse effects in DSS-induced colitis and inhibition of LPS-induced NLRP3 inflammasome activation in macrophages

Activation of inflammasomes particularly NLRP3 and NLRP1, plays central role in driving inflammatory response associated with inflammatory bowel disease (IBD). The anti-inflammatory and the antioxidant's efficacy of TXM peptides was evaluated using the tetra peptides TXM-CB13 both *in-vivo*, in a dextran sulfate sodium (DSS)-induced colitis mouse model, and in vitro*,* in LPS-induced NLRP3 inflammasome activation in RAW264.7 macrophages (Table II) [65]. TXM-CB13 suppressed both protein and mRNA levels of NLRP3, Mlck, and IL-1β in colonic tissues. In contrast it upregulated the expression of the intestinal barrier proteins Occludin and ZO-1, crucial for tight junctions formation and permeability regulation of epithelial and endothelial barriers. TXM-CB13 also increased the expression of NLRP1, an inflammasome sensor, as demonstrated by immunohistochemistry and Western blot analysis. These effects contributed to the restoration of mucosal barrier integrity and attenuation of DSS-induced colitis. *In vitro*, TXM-CB13 inhibited LPS-induced TLR4 signaling, and through reducing MyD88 expression triggered significant decrease in NF-κB pathway activation. Additionally, TXM-CB13 attenuated activation of the NLRP3 inflammasome complex, as indicated by reduced Gasdermin D, and IL-1β, and prevented the ROS-mediated dissociation of TXNIP from Trx1 [65,66]. These results are consistent with previous findings demonstrating that TXM-CB3 prevents expression of TXNIP/TBP-2 in the brains of the leptin-receptor-deficient Zucker diabetic fatty (ZDF) rats ([18]; see Section 3.2). The **protective effects of TXM-CB13** in both **DSS-induced colitis in mice** and **LPS-induced NLRP3 activation in RAW264.7 macrophages** highlights a promising dual anti-inflammatory and antioxidant mechanisms, indicating its potential as a therapeutic candidate for inflammatory bowel disease.

### 3.10 Reduction of epilepsy seizure frequency and prolongation of seizure latency in epilepsy

Epilepsy is a chronic neurological disorder marked by recurrent seizures, with growing evidence implicating OS and neuroinflammation in its pathogenesis and progression. Despite the availability of a large variety of anti-seizure medications, approximately 30–40 % of patients remain refractory to treatment due to disease progression and pharmaco-resistance, which underscore critical need for novel therapeutic interventions [67,68].

The proposed use of antioxidant treatment as a strategy to target multiple epileptogenic processes by preventing OS-induced inflammation in neurons [69], led to evaluate the anti-inflammatory and the antioxidant's efficacy of TXM peptides as potential treatment for epilepsy. In *in vivo* studies, Sing et al., showed that TXM-CB3 protected epilepsy-associated cognitive deficits, and significantly reduced seizure frequency, prolonged seizure latency, and mitigated cumulative seizure burden [Sing, 2025].

https://papers.ssrn.com/sol3/papers.cfm?abstract_id=5212378. Neuroprotection was conferred by i) preserving hippocampal neuronal integrity, ii) attenuating oxidative-induced DNA/RNA damage, and iii) ameliorating spatial memory deficits and anxiety-like behaviors. During the chronic phase of epilepsy protection by TXM-CB3 treatment did not alter locomotion or recognition memory, indicating its targeted efficacy without compromising general cognitive or motor functions [Sing 2025]. In vitro, TXM-CB3 effectively suppressed ROS production in a model of epileptiform activity. It also reduced the expression of pro-inflammatory cytokines (TNF-α, IL-6, IL-1β) (see Section 3.5 and Section 3.7), and enhanced he expression of anti-inflammatory IL-10 expression during epileptiform activity.

## 4 In vivo and in vitro studies using SuperDopa (SD), a TXM-peptide derivative, demonstrated its dual antioxidant and dopaminergic activities

### 4.1 Cognitive restoration in the rotenone rat model of Parkinson's disease

The deterioration of dopaminergic neurons and the progression of PD is closely associated with OS-induced degenerative processes such as mitochondrial dysfunction, excitotoxicity, nitric oxide toxicity, and neuroinflammation. The current gold standard for PD treatment is replenishment of dopamine levels in the substantia nigra pars compacta (SNpc) through the administration of levodopa. While this approach offers symptomatic relief during an initial “honeymoon” period of approximately 4–5 years, it does not halt the underlying neurodegenerative process, and disease progression continues.

The broad redox-protective properties of TXM peptides in models of OS and inflammation-associated disorders prompted the design of a TXM-like compound that combines intrinsic redox activity with levodopa delivery (Table II). This TXM-derivative termed SuperDopa (SD) (Fig. 3B *left*), was developed as a disease-modifying strategy for PD aimed at enhancing dopaminergic neuron survival. SD integrates the antioxidant properties of NAC with the symptomatic benefits of levodopa within a single molecule. Unlike NAC, a key advantage of SD lies in its ability to cross the BBB, effectively acting as a brain-permeable carrier for NAC and delivering antioxidant protection directly to the central nervous system. Upon proteolysis, SD can potentially generate 8 levodopa containing fragments, and like other TXM-derived tri-peptides, up to 10 distinct Cys-containing redox-active fragments (Fig. 3B, *right*). This unique cluster of redox-active and levodopa entities may act synergistically to enhance and amplify the protective effect on dopaminergic neurons.

SD was tested in vivo using the acute rotenone rat model of PD to evaluate its ability to reverse cognitive impairment. In these studies, i.p injection of SD showed a significant relief of motor disabilities, demonstrated by the rotarod behavior test, the rearing cylinder test, and the beam-walk test [70]. In parallel, in vitro studies demonstrated that SD reduced rotenone-induced α-synuclein (α-Syn) accumulation in human SH-SY5Y neuronal cells and reversed OS–induced phosphorylation of JNK and p38^MAPK^. The attenuation of this MAPK-driven inflammatory and apoptotic signaling in vitro, alongside the protection against rotenone-induced motor impairment in vivo, reflects the dual antioxidant and anti-inflammatory activities of SD [70]. It was hypothesized that upon entry into dopaminergic neurons SD undergoes proteolytic cleavage, simultaneously releasing levodopa along with redox-active NAC and Cys residues to counteract OS and inflammation. This integrated therapeutic approach joined in a single molecule, positions SD as both a levodopa precursor and a redox-active agent, uniquely capable of delivering NAC across the BBB. As such, SD holds strong potential as a disease-modifying therapy for slowing the progression of PD and provide relief beyond the symptomatic conventional levodopa therapy.

### 4.2 Reversal of photic and-nonphotic stress in ARPE-15 cells

The development and progression of retinal associated diseases such as retinitis pigmentosa, geographical atrophy, age-related macular degeneration (AMD), myopia, and others are closely linked to the OS and chronic inflammation in the retinal pigment epithelium (RPE). The well-being of RPE cells is critically dependent on levodopa which serves as the endogenous ligand of the G-protein-coupled receptor GPR143 [71]. Activation of GPR143 receptor expressed in the apical surface of RPE cells by levodopa has been shown to upregulate the pigment epithelium-derived factor (PEDF), a potent anti-angiogenic molecule that in turn suppresses the production of vascular endothelial growth factor (VEGF) [72,73] (Table II). These results indicate the potential of levodopa as an anti-VEGF therapeutic approach for neovascular AMD [74,75].

Given the established role of levodopa in supporting RPE function, the effects of SD and other TXM-peptides were tested for their efficacy in reversing OS-induced damage in ARPE-19 cells, a spontaneously arising human RPE cell line commonly used to study retinal function in vitro. The redox-modulating activities of the thiol-based peptides TXM-CB30, TXM-CB13, and SD, as well as the NAC derivative AD4/NACA, were evaluated by Olchawa et al. [36] (see section 2.8). Cellular protection by SD was correlated with the compounds’ abilities to scavenge DPPH radicals, quench singlet oxygen, and exert anti-inflammatory activity through inhibition of the MAPK signaling pathway. Compared to GSH, the levodopa derivative SD exhibited approximately two-fold higher bimolecular rate constant for singlet oxygen quenching in aqueous solution, indicating superior reactivity relative to TXM-CB30 and TXM-CB13 [36]. Furthermore, its ability to inhibit AuF-induced activation of MAPKs, specifically JNK1/2 and ERK1/2, further confirmed the potent antioxidant and anti-inflammatory properties of the thiol-levodopa peptide. These findings suggest a therapeutic potential for TXM-peptides in the treatment of OS-related retinal disorders.

## 5 Summary

The **TXM peptides** are a novel family of low-molecular-weight tri- and tetrapeptides designed to reproduce the redox functions of **Trx1** and **Trx2**. They incorporate the canonical thioredoxin active-site motifs (–Cys-X-X-Cys– and –Cys-X-Cys–), each containing two thiol groups that confer strong reducing capacity. TXM peptides appear to mimic Trx1 activity by sustaining a reducing intracellular environment in the cytosol, and indirectly mimic Trx2 activity following their penetration into mitochondria.

Functionally, TXM peptides potentiate cellular antioxidant defenses by lowering oxidant levels, regulating ROS levels, preventing OS, repairing oxidative damage, and inhibiting inflammatory and apoptotic signaling. Their mechanisms include acting as **GSH precursors** through Cys generation, catalyzing S-denitrosylation, **chelating redox-active metals** (zinc and copper), and **modulating redox-sensitive signaling cascades**.

In experimental models, TXM peptides have demonstrated protective efficacy mitigating **redox-associated cognitive disorders** (e.g., mild traumatic brain injury, rotenone-induced toxicity), lowering cytokine levels and NF-kB, attenuating allergic airway disease, restoring catecholamine and insulin release, and improving systolic cardiac function, among others. Due to their peptide nature, they are likely hydrolyzed into amino acids, suggesting a **safety and non-toxicity profile**.

Overall, TXM peptides represent promising therapeutic candidates capable of downregulating inflammatory signaling, protecting cells from oxidative injury, and modulating redox homeostasis.

## Declaration of competing interest

The authors declare that they have no known competing financial interests or personal relationships that could have appeared to influence the work reported in this paper.

## Data Availability

No data was used for the research described in the article.
